# Small RNAs Prevent Transcription-Coupled Loss of Histone H3 Lysine 9 Methylation in *Arabidopsis thaliana*

**DOI:** 10.1371/journal.pgen.1002350

**Published:** 2011-10-27

**Authors:** Raymond A. Enke, Zhicheng Dong, Judith Bender

**Affiliations:** 1 Wilmer Eye Institute, Department of Ophthalmology, Johns Hopkins University School of Medicine, Baltimore, Maryland, United States of America; 2 Department of Molecular Biology, Cell Biology, and Biochemistry, Brown University, Providence, Rhode Island, United States of America; The University of North Carolina at Chapel Hill, United States of America

## Abstract

In eukaryotes, histone H3 lysine 9 methylation (H3K9me) mediates silencing of invasive sequences to prevent deleterious consequences including the expression of aberrant gene products and mobilization of transposons. In *Arabidopsis thaliana*, H3K9me maintained by SUVH histone methyltransferases (MTases) is associated with cytosine methylation (5meC) maintained by the CMT3 cytosine MTase. The SUVHs contain a 5meC binding domain and CMT3 contains an H3K9me binding domain, suggesting that the SUVH/CMT3 pathway involves an amplification loop between H3K9me and 5meC. However, at loci subject to read-through transcription, the stability of the H3K9me/5meC loop requires a mechanism to counteract transcription-coupled loss of H3K9me. Here we use the duplicated *PAI* genes, which stably maintain SUVH-dependent H3K9me and CMT3-dependent 5meC despite read-through transcription, to show that when *PAI* sRNAs are depleted by dicer ribonuclease mutations, *PAI* H3K9me and 5meC levels are reduced and remaining *PAI* 5meC is destabilized upon inbreeding. The dicer mutations confer weaker reductions in *PAI* 5meC levels but similar or stronger reductions in *PAI* H3K9me levels compared to a *cmt3* mutation. This comparison indicates a connection between sRNAs and maintenance of H3K9me independent of CMT3 function. The dicer mutations reduce *PAI* H3K9me and 5meC levels through a distinct mechanism from the known role of dicer-dependent sRNAs in guiding the DRM2 cytosine MTase because the *PAI* genes maintain H3K9me and 5meC at levels similar to wild type in a *drm2* mutant. Our results support a new role for sRNAs in plants to prevent transcription-coupled loss of H3K9me.

## Author Summary

Methylation of histone H3 at the lysine 9 position (H3K9me) is a fundamental chromatin modification that suppresses expression from invasive and repetitive sequences such as transposons. In plant genomes, regions modified by H3K9me are maintained with precise boundaries. However, at junctions where H3K9me target regions are subject to read-through transcription from outside promoters, the stability of H3K9me patterns is jeopardized by transcription-coupled processes that remove this modification. We show that maintenance of H3K9me patterns at such vulnerable sites requires small RNAs corresponding to the H3K9me target region. We use a sensitive reporter system to show that, in the absence of small RNAs, target regions subject to read-through transcription undergo an immediate reduction in H3K9me levels, followed by further losses in progeny plants upon inbreeding. Our results support a new function for small RNAs in maintaining accurate H3K9me patterns in the plant genome.

## Introduction

The eukaryotic cell is under constant threat from transposons and other invasive sequences. Transposons can drain cellular resources for RNA and protein synthesis and can damage the cell through expression of aberrant gene products or activation of transposon movement. A major mechanism to protect against these deleterious effects is to target transposons and other repetitive sequences for silencing mediated through chromatin modifications. In most eukaryotes, transposon chromatin is marked by methylation of histone H3 at the lysine 9 position (H3K9me). In some eukaryotes including mammals and plants transposon chromatin is also marked by cytosine methylation (5meC). An important question is how H3K9me and 5meC are accurately maintained on transposons but not on host genes.

A conserved strategy to maintain H3K9me and 5meC is to use the modifications as methyltransferase (MTase) binding recognition motifs. For example, in *Arabidopsis thaliana*, dimethylation of H3K9 (H3K9me2) maintained by three partially redundant histone MTases—SUVH4 (also known as KYP, At5g13960), SUVH5 (At2g35160), and SUVH6 (At2g22740)—is associated with 5meC maintained by the CMT3 cytosine MTase (At1g69770) [1]–[4]. The SUVH MTases contain a 5meC binding domain and CMT3 contains an H3K9me binding domain, suggesting that the SUVH/CMT3 pathway involves an amplification loop that can perpetuate both H3K9me and 5meC [5], [6]. Consistent with this model, mutations in the CMT3 or MET1 (At5g49160) cytosine MTases, which act to maintain 5meC in non-CG and CG sequence contexts respectively, result in reduced H3K9me2 levels on transposons and repetitive sequences [3], [7]–[10]. In addition, a *suvh4 suvh5 suvh6* triple H3 K9 MTase mutant displays similar reduced non-CG methylation patterns to a *cmt3* mutant [4]. Although the H3K9me/5meC amplification loop provides a mechanism to stably maintain both modifications in untranscribed regions of the genome, at junctions where modified sequences are transcribed through from nearby unmodified promoters, H3K9me can be removed by transcription-associated histone replacement or histone demethylation [7], [11]. What prevents transcriptional destabilization of H3K9me patterns?

Duplicated Arabidopsis genes encoding the tryptophan synthesis enzyme phosphoribosylanthranilate isomerase (PAI) provide an ideal system to understand the balance between transcription and SUVH-mediated H3K9me2/CMT3-mediated 5meC. In most Arabidopsis strains there are three unlinked *PAI* gene duplications that lack 5meC [12]. However, in the Wassilewskija (Ws) strain one of the *PAI* loci is rearranged as a tail-to-tail inverted repeat (IR) of two genes *PAI1–PAI4* (At1g07780), which triggers the recognition of *PAI* sequences as invaders. The *PAI1–PAI4* IR as well as two unlinked singlet genes *PAI2* (At5g05590) and *PAI3* (At1g29410) are modified by H3K9me2 and 5meC, coextensive with their regions of shared sequence identity [3], [13] (see Figure S1 for *PAI* gene maps). The *PAI1–PAI4* IR is fused to a heterologous promoter with a transcription start site approximately 500 base pairs (bp) upstream of the *PAI1* 5meC boundary, which drives constitutive expression of *PAI1* transcripts [14]. The polyadenylated transcripts that accumulate from this locus consist of a majority class that terminates normally in the *PAI1* 3′ untranslated region at the center of the IR and a minority class that extends through *PAI1* into palindromic *PAI4* sequences to provide a source of fold-back double-stranded RNA (dsRNA). Therefore the *PAI1–PAI4* locus is able to stably maintain H3K9me2 and 5meC on the IR sequences even in the face of substantial read-through transcription. The *PAI2* and *PAI3* singlet genes also stably maintain H3K9me2 and 5meC even though they are likely to be only partially silenced by limited upstream modifications: at *PAI2* 5meC extends only 250 bp upstream of the predicted transcription start site, and at *PAI3* 5meC extends only as far as the predicted transcription start site [12], [13].

Arabidopsis uses three cytosine MTase pathways to control 5meC: the CMT3 pathway maintains 5meC mainly in non-CG contexts in conjunction with the SUVH H3K9 MTases, the MET1 pathway maintains 5meC mainly in CG contexts, and the DRM2 (At5g14620) pathway initiates 5meC on new invasive sequences under the guidance of small RNAs (sRNAs), as well as contributing to maintenance of non-CG methylation at some loci [15]. In a *cmt3* or a *suvh4 suvh5 suvh6* mutant, the Ws *PAI* genes are depleted for 5meC in non-CG contexts [4], [16]. In addition, in a *cmt3 met1* double mutant the *PAI* genes are depleted for 5meC in all contexts [3]. Therefore, the DRM2 pathway plays a minimal role in the maintenance of *PAI* 5meC patterns. However, genetic or epigenetic changes that impair the production of transcripts that read through from *PAI1* into palindromic *PAI4* sequences at the *PAI1–PAI4* IR cause reduced levels of *PAI* 5meC in non-CG contexts [13], [14], [17], [18]. In light of these results, we hypothesized that sRNAs processed from dsRNAs might underlie a mechanism to prevent the loss of SUVH/CMT3-mediated modifications due to read-through transcription, independently of the role for sRNAs in guiding DRM2.

To test the hypothesis that sRNAs control the SUVH/CMT3 pathway, we used mutations in Arabidopsis dicer-like (DCL) ribonucleases to block processing of sRNAs from dsRNAs, and monitored the effects on Ws *PAI* gene H3K9me2 and 5meC levels. Arabidopsis encodes four DCLs (reviewed in [19]). DCL1 (At1g01040) is specialized for processing 21 nucleotide (nt) microRNAs (miRNAs) needed for developmental gene regulation, whereas DCL2 (At3g03300), DCL3 (At3g43920), and DCL4 (At5g20320) have partially redundant roles in processing sRNAs used in other silencing pathways. For example, DCL3 processes 24 nt sRNAs used to guide DRM2 to matching target sequences such as transgene insertions and transposons [20], [21]. In a *dcl3* mutant DCL2 and DCL4 can partially compensate by processing 22 nt and 21 nt sRNAs respectively corresponding to the same genomic target sequences [22], [23].

Here we show that the *dcl2 dcl3 dcl4* mutant has reduced levels of H3K9me2 and non-CG methylation on *PAI* sequences relative to wild type, corresponding to loss of *PAI* sRNAs. We also show that a *drm2* mutant maintains similar levels of *PAI* H3K9me2 and 5meC relative to wild type. Therefore the *PAI* genes illustrate that DCL-dependent sRNAs help maintain SUVH/CMT3-mediated modifications through a distinct mechanism from their role in guiding DRM2. In the *dcl* mutant there is a weaker reduction in *PAI* 5meC levels but a similar or stronger reduction in *PAI* H3K9me2 levels compared to a *cmt3* mutant, indicating a connection between sRNAs and maintenance of H3K9me2 patterns independent of CMT3 function. We also show that upon inbreeding in the absence of DCL function, remaining *PAI* 5meC is destabilized. Our results reveal a new pathway for sRNA control of H3K9me2 and associated 5meC patterns in plants. This pathway provides a homeostatic mechanism to use a product of read-through transcription—sRNAs—as a means to counteract transcription-coupled loss of H3K9me2 on transposons and repeats.

## Results

### Dicer mutations cause reduced levels of *PAI* non-CG methylation

To test whether dicer-dependent sRNAs contribute to maintenance of *PAI* 5meC patterns, we generated strains where dicer mutations were combined with the three methylated *PAI* loci from Ws and assayed *PAI* 5meC patterns using both DNA gel blot and bisulfite sequencing assays. For *PAI* DNA gel blot analysis we cleaved genomic DNA with each of three 5meC-sensitive restriction enzymes that have cleavage sites within methylated *PAI* sequences: *Hin*cII (sensitive to methylation of the outermost non-CG cytosines in 5′ atGTCAACag 3′, where the recognition sequence is shown in uppercase), *Msp*I (sensitive to methylation of the outer non-CG cytosines in 5′ CCGG 3′, and *Hpa*II (sensitive to methylation of either the inner CG or outer non-CG cytosines in 5′ CCGG 3′). *Hin*cII cleaves at the translational start codons of *PAI1*, *PAI2* and *PAI4* but not *PAI3*, and the *Msp*I/*Hpa*II isoschizomers cleave in the second introns of *PAI2*, *PAI3*, and *PAI4* but not *PAI1* (Figure S1).

**Figure 1:**
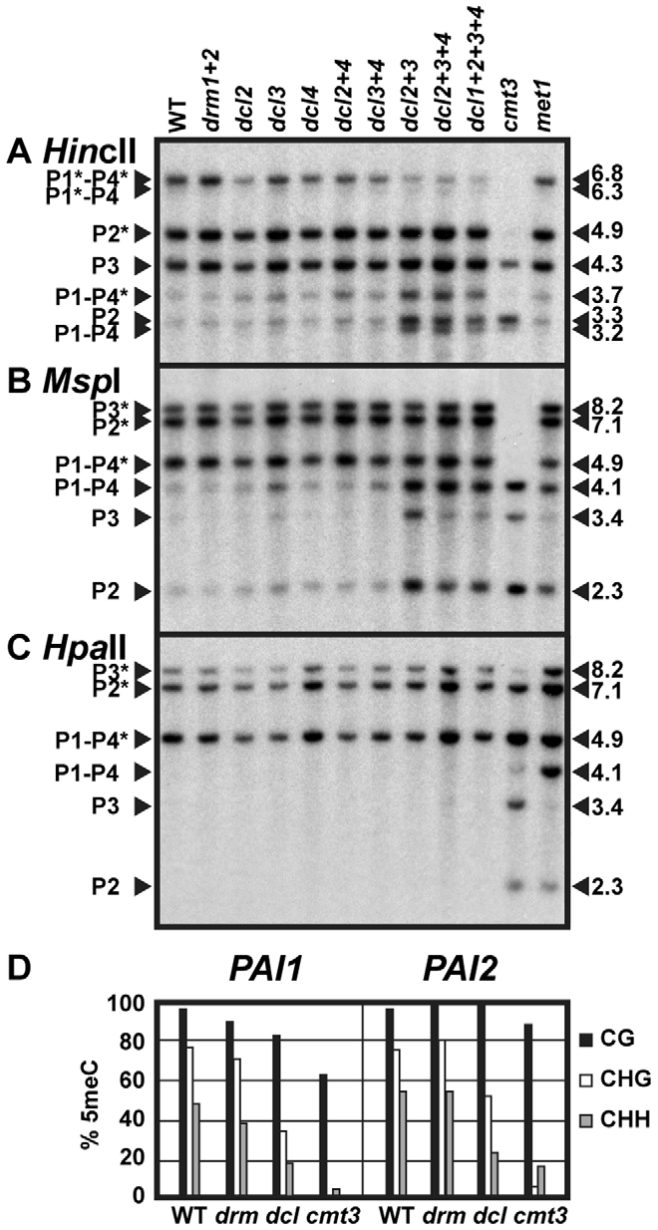
*PAI* non-CG methylation levels are reduced in *dcl* mutants. (A), (B), and (C) DNA gel blot assays for *PAI* 5meC. Genomic DNA from the indicated strains was cleaved with (A) *Hin*cII, (B) *Msp*I, or (C) *Hpa*II (isoschizomer of *Msp*I) and used in DNA gel blot analysis with a *PAI* cDNA probe [17]. *PAI* restriction maps are shown in Figure S1. P1–P4 indicates *PAI1–PAI4*, P2 indicates *PAI2*, and P3 indicates *PAI3*, with bands diagnostic of 5meC on *PAI*-internal sites marked with asterisks. Lengths of cleaved fragments in kilobases (kb) are indicated along the right margins. Multiple mutant *drm* or *dcl* combinations are indicated with a + sign separating each mutation; for example, *drm1+2* indicates a *drm1 drm2* mutant and *dcl2+3* indicates a *dcl2 dcl3* mutant. WT indicates wild type Ws. (D) Bisulfite sequencing of *PAI1* and *PAI2*. Genomic DNA from the indicated strains was treated with sodium bisulfite and used as a template for PCR amplification of the top strands of the *PAI1* or *PAI2* proximal promoter/first exon regions. Eight independent clones were sequenced for each locus. The percentage of 5meC out of total cytosines sequenced within the region of *PAI* sequence identity (344 bp for *PAI1* or 338 bp for *PAI2*) is shown, divided into the contexts CG (black), CHG (white), or CHH (gray). For comparison, previously determined patterns for wild type Ws [13] and Ws *cmt3* [16] are shown. *drm* indicates the *drm1 drm2* mutant, and *dcl* indicates the *dcl2 dcl3 dcl4* mutant.

We found that genomic DNA prepared from *dcl2*, *dcl3*, and *dcl4* single insertional null mutants and the *dcl2 dcl4* and *dcl3 dcl4* double mutants had similar *PAI* cleavage patterns to wild type Ws genomic DNA when assessed by *Hin*cII, *Msp*I, or *Hpa*II DNA gel blot assays (Figure 1). In contrast, genomic DNA prepared from the *dcl2 dcl3* and *dcl2 dcl3 dcl4* mutants displayed increased cleavage with *Hin*cII at *PAI1–PAI4* and *PAI2*, and with *Msp*I at *PAI1–PAI4*, *PAI2*, and *PAI3* relative to wild type Ws, diagnostic of partially reduced non-CG methylation levels at all three *PAI* loci. Bisulfite sequencing of *PAI1* and *PAI2* proximal promoter/first exon regions in the *dcl2 dcl3 dcl4* mutant compared to wild type Ws and Ws *cmt3* showed that there was a partial loss of 5meC in CHG and CHH contexts. Therefore, the bisulfite sequencing data are consistent with the DNA gel blot assays. The results indicate that DCL2 and DCL3 act redundantly to maintain *PAI* non-CG methylation patterns.

To determine whether the miRNA processing dicer DCL1 contributes to the remaining *PAI* non-CG methylation in the *dcl2 dcl3 dcl4* triple mutant relative to *cmt3*, we included a *dcl1 dcl2 dcl3 dcl4* quadruple mutant strain in the DNA gel blot analysis (Figure 1). Because *dcl1* null alleles are embryo-lethal we used a partial-function *dcl1-9* allele that is viable but female-sterile [24], [25]. The *dcl1 dcl2 dcl3 dcl4* mutant displayed similar cleavage patterns to the *dcl2 dcl3 dcl4* mutant, indicating that the *dcl1-9* mutation does not enhance the partial loss of 5meC conferred by mutation of the other three *DCL* genes. In subsequent studies we focused on the *dcl2 dcl3 dcl4* mutant, which has global depletion of sRNAs other than miRNAs [23].

For comparison to the *dcl* mutants we included genomic DNA prepared from cytosine MTase mutants in the Ws background (Figure 1). DRM2 is the major cytosine MTase controlling initiation of 5meC, but the related DRM1 MTase (At5g15380) could also contribute to this pathway [26]. Therefore we used a *drm1 drm2* double null insertional mutant. DNA from the Ws *drm1 drm2* mutant displayed similar *PAI* cleavage patterns to wild type Ws in all three DNA gel blot assays, and similar 5meC patterns to wild type Ws in bisulfite sequencing analysis of *PAI1* and *PAI2* proximal promoter regions. DNA from a Ws *met1* mutant displayed increased cleavage at all three *PAI* loci with *Hpa*II, and partially increased cleavage at *PAI1–PAI4* and *PAI2* with *Msp*I, but no difference from wild type *PAI* cleavage patterns with *Hin*cII in DNA gel blot assays, diagnostic of a partial loss of 5meC in CG and CCG contexts. DNA from the *cmt3* mutant displayed nearly complete cleavage with *Hin*cII and *Msp*I, and partially increased cleavage with *Hpa*II in DNA gel blot assays, diagnostic of strong loss of 5meC mainly in CHG and CHH contexts. Compared to the cytosine MTase mutants across the three DNA gel blot assays, the *dcl* mutant *PAI* demethylation phenotypes are consistent with a partial defect in the SUVH/CMT3 pathway rather than a defect in the DRM or MET1 pathways.

DCL3 and DRM2 are key factors in establishing new 5meC imprints. We used a previously developed genetic assay combining the Ws *PAI* IR dsRNA source locus with an unmethylated *PAI2* target gene from another strain background to show that *dcl3* and *drm1 drm2* mutations impair the acquisition of new 5meC on *PAI2* (Text S1, Figure S2). Therefore the *PAI* genes use the same DCL3/DRM pathway for establishing 5meC imprints as other characterized loci. However, once *PAI* 5meC patterns are established, the DCL3/DRM pathway plays a minimal role in long-term maintenance (Figure 1).

### The *dcl2 dcl3 dcl4* mutant has reduced levels of *PAI* H3K9me2

We used chromatin immunoprecipitation (ChIP) analysis with H3K9me2-specific antibodies on chromatin prepared from the *dcl2 dcl3 dcl4* mutant compared to chromatin prepared from wild type, *suvh4 suvh5 suvh6*, *cmt3*, *drm1 drm2*, or *cmt3 drm1 drm2* strains to determine whether the *dcl* mutations affect levels of H3K9me2 as well as non-CG methylation on *PAI* sequences. Chromatin was analyzed by quantitative PCR with primer pairs specific for the *PAI1* arm of the *PAI1–PAI4* IR locus or the *PAI2* singlet gene.

**Figure 2:**
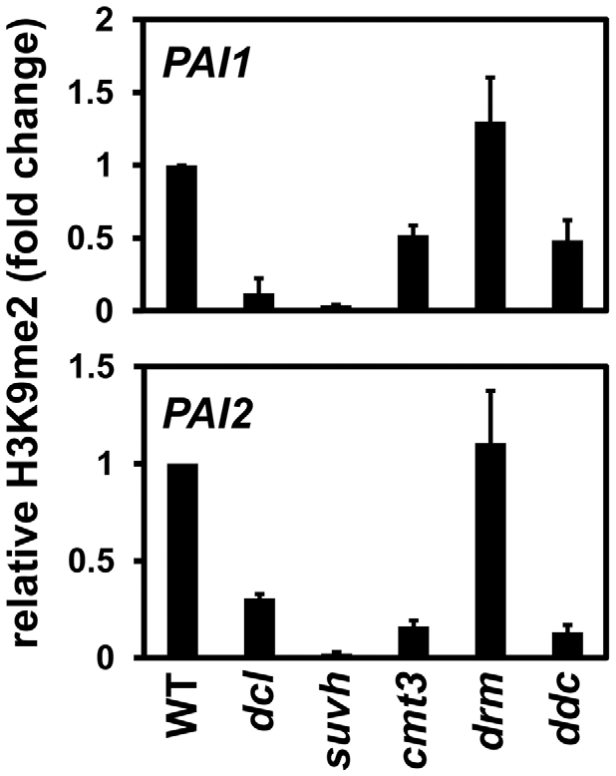
*PAI1* and *PAI2* H3K9me2 levels are reduced in the *dcl2 dcl3 dcl4* mutant. Primers specific for *PAI1* or *PAI2* were used for quantitative PCR amplification from total input chromatin or chromatin immunoprecipitated with antibodies specific for H3K9me2 from the indicated strains. WT indicates wild type Ws, *dcl* indicates the *dcl2 dcl3 dcl4* mutant, *suvh* indicates the *suvh4 suvh5 suvh6* mutant, *drm* indicates the *drm1 drm2* mutant, and *ddc* indicates the *drm1 drm2 cmt3* mutant. PCR results are displayed as fold change relative to WT. Results were reproduced in three biological replicates with error bars representing standard error among biological replicates.

At both *PAI1–PAI4* and *PAI2* the *dcl2 dcl3 dcl4* mutant had reduced levels of H3K9me2 relative to wild type, although not as strongly as in the *suvh4 suvh5 suvh6* H3K9 MTase mutant (Figure 2). Comparing the ChIP results to the assays for 5meC (Figure 1), the reduced *PAI* H3K9me2 levels in the *dcl2 dcl3 dcl4* mutant are still sufficient to support substantial CMT3 activity. Therefore CMT3 might be able to use even sparsely distributed H3K9me2 as a localization signal.

At both *PAI* loci the *cmt3* mutant also had partially reduced levels of H3K9me2, presumably because reduced 5meC levels impair SUVH localization to *PAI* sequences. At *PAI2* increased transcription due to proximal promoter demethylation in a *cmt3* mutant could also contribute to reduced H3K9me2 levels, perhaps accounting for a stronger relative reduction at *PAI2* than at *PAI1–PAI4* [3], [16]. The *dcl2 dcl3 dcl4* mutant had similar reduction in H3K9me2 levels to the *cmt3* mutant at *PAI2*, but a stronger reduction at *PAI1–PAI4*. In contrast, the *dcl2 dcl3 dcl4* mutant had weaker reductions in *PAI2* and *PAI1–PAI4* non-CG methylation levels compared to *cmt3* (Figure 1). This comparison indicates that the *dcl2 dcl3 dcl4* mutations impair maintenance of H3K9me2 independently of effects on CMT3 function. If the *dcl* mutations acted by impairing CMT3 to cause a partial reduction in *PAI* 5meC levels as the primary consequence, then the resulting reduction in H3K9me2 levels would be expected to be less than in the *cmt3* mutant.

At both *PAI1–PAI4* and *PAI2*, the *drm1 drm2* mutant displayed similar levels of H3K9me2 to wild type, and the *drm1 drm2 cmt3* mutant displayed similar levels of H3K9me2 to *cmt3* (Figure 2). Therefore, the DRM cytosine MTases do not contribute to maintenance of *PAI* H3K9me2 patterns.

### The *dcl2 dcl3 dcl4* mutant is depleted for *PAI* sRNAs

**Figure 3:**
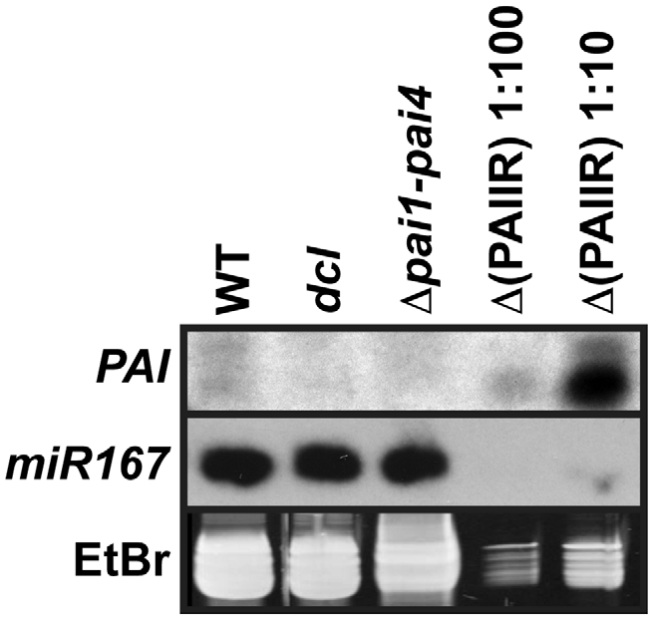
The *dcl*2 *dcl3 dcl4* mutant is depleted for *PAI* sRNAs. sRNA fractions from the indicated strains were used in RNA gel blot analysis with a 35 nt LNA oligonucleotide probe antisense to the *PAI1* fifth exon sequence (*PAI*, top panel). A replicate blot was probed with a 21 nt oligonucleotide antisense to *miR167* (middle panel). The low molecular weight RNA portion of the ethidium bromide (EtBr) stained gel, representing 5S RNA and tRNA, is shown prior to transfer of RNA to a membrane (bottom panel). WT indicates wild type Ws, *dcl* indicates the *dcl2 dcl3 dcl4* mutant, and Δ(PAIIR) indicates *Δpai1–pai4* transformed with the *PAIIR* transgene [14]. The Δ(PAIIR) RNA was loaded at ten-fold (1∶10) and a hundred-fold (1∶100) dilutions relative to the other samples.

To determine whether reduced *PAI* non-CG methylation and H3K9me2 levels in *dcl2 dcl3 dcl4* correlate with loss of *PAI* sRNAs, we used RNA gel blot analysis to detect *PAI* sRNAs (Figure 3). As a negative control we used a mutant derivative of Ws, *Δpai1–pai4*, where the *PAI1–PAI4* IR source of dsRNA DCL substrates has been deleted by homologous recombination between flanking direct repeat sequences [17]. As a positive control we used the *Δpai1–pai4* strain transformed with a *PAIIR* transgene consisting of an IR of approximately 700 bp of *PAI* cDNA sequences transcribed by the strong constitutive Cauliflower Mosaic Virus 35S promoter [14]. We previously determined that *PAI* sRNAs could be detected in the *Δpai1–pai4*(*PAIIR*) transgenic strain but not in wild type Ws using RNA gel blot analysis with a *PAI* cDNA riboprobe. Furthermore, high-throughput sRNA sequencing in the C24 strain that has a similar *PAI1–PAI4* IR to Ws detected *PAI* sRNAs at low levels [27]. We therefore optimized detection of rare *PAI* sRNAs by designing a high-affinity locked nucleic acid (LNA) probe corresponding to the sense strand of a 35 nt sequence in the *PAI* fifth exon.

In wild type Ws the LNA probe detected low levels of *PAI* sRNA species of both shorter and longer sizes, between 21 and 24 nt relative to size markers, above the background signal in the *Δpai1–pai4* negative control strain (Figure 3). This pattern is consistent with processing of *PAI1–PAI4* palindromic transcripts into sRNAs by more than one dicer. Correspondingly, the C24 high-throughput sequencing analysis detected *PAI* sRNAs between 21 and 24 nt long covering the entire IR region [27]. The wild type Ws levels of endogenous *PAI* sRNAs were comparable to levels detected in a hundred-fold dilution of RNA prepared from the *Δpai1–pai4*(*PAIIR*) positive control transgenic strain (Figure 3). The transgenic strain produced mostly smaller *PAI* sRNAs, as previously observed for this strain using a *PAI* cDNA riboprobe [14], presumably due to differences in *PAI* IR expression patterns and structure.

*PAI* sRNA species were depleted in the *dcl2 dcl3 dcl4* strain to similar background levels as detected in *Δpai1–pai4* (Figure 3). This result supports the hypothesis that loss of *PAI* sRNAs underlies the reduction in SUVH-dependent H3K9me2 and CMT3-dependent 5meC on *PAI* sequences. The lack of residual *PAI* sRNAs in *dcl2 dcl3 dcl4* indicates a minimal contribution of DCL1 to generating these species, although DCL1 is functional in processing microRNAs such as *miR167*.

### The *dcl2 dcl3 dcl4* mutant has destabilized *PAI2* silencing and 5meC

To determine whether loss of sRNAs causes destabilization of remaining *PAI* silencing modifications upon inbreeding by self-pollination, we introduced the *dcl2 dcl3 dcl4* mutations into a Ws *pai1* reporter background where silencing of the *PAI2* singlet gene can be monitored by visual inspection. In wild type Ws, *PAI1* expressed from the heterologous upstream promoter is the major source of PAI enzyme; expression of *PAI2* is impaired by H3K9me2/5meC on proximal promoter sequences and *PAI3* and *PAI4* do not encode functional enzyme due to polymorphisms [12]. In the Ws *pai1* missense mutant, the impairment of *PAI2* expression is revealed through tryptophan deficiency phenotypes including reduced size and blue fluorescence under ultraviolet (UV) light caused by accumulation of the tryptophan precursor anthranilate [28]. The stable maintenance of *PAI2* H3K9me2/5meC in *pai1* is reflected in stable maintenance of blue fluorescence across generations of inbreeding. Mutations that decrease *PAI2* H3K9me2 and/or 5meC levels in the Ws *pai1* background, including *cmt3*, *met1*, and *suvh4*, result in reduced fluorescence [2], [16], [28].

**Figure 4:**
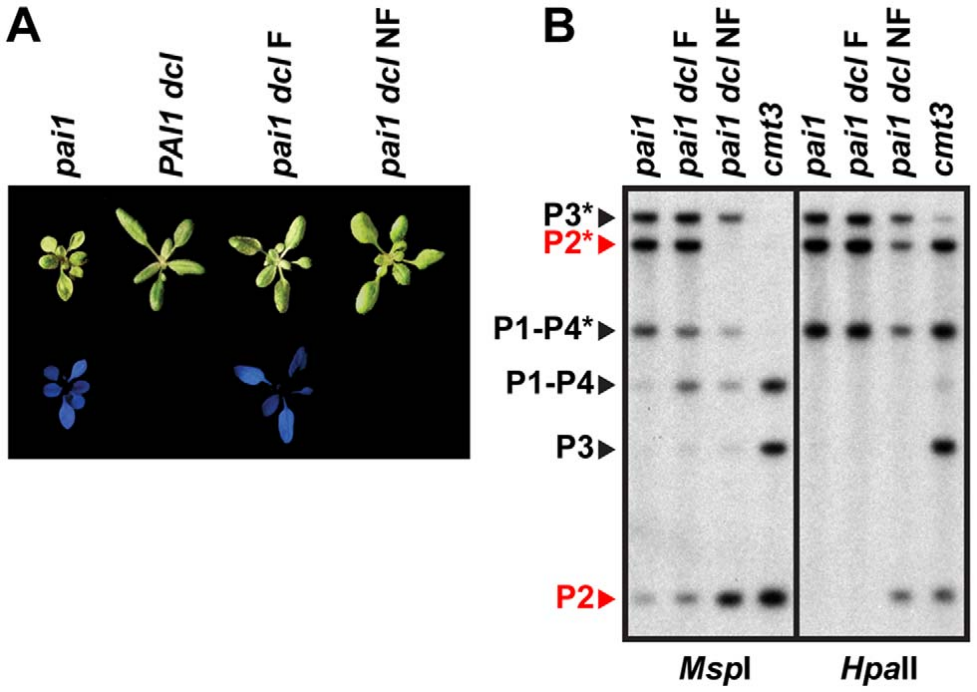
*PAI2* 5meC is destabilized in *pai1 dcl2 dcl3 dcl4*. (A) Blue fluorescence diagnostic of *PAI2* silencing in the *pai1* background. Representative 2.5-week-old plants of the indicated strains are shown under visible (top) or UV (bottom) light. (B) DNA gel blot assays for *PAI* 5meC. Genomic DNA from the indicated strains was cleaved with *Msp*I or *Hpa*II isoschizomers and used in DNA gel blot analysis with a *PAI* cDNA probe. P1–P4 indicates *PAI1–PAI4*, P2 indicates *PAI2*, and P3 indicates *PAI3*, with bands diagnostic of 5meC on *PAI*-internal sites marked with asterisks. Bands corresponding to *PAI2*, which controls the fluorescence phenotype, are highlighted in red. Molecular weights of fragments are as shown in Figure 1. *dcl* indicates the *dcl2 dcl3 dcl4* mutant, F indicates fluorescent, and NF indicates non-fluorescent.

The initial *pai1 dcl2 dcl3 dcl4* strain displayed partially reduced *PAI* 5meC patterns similar to the *PAI1 dcl2 dcl3 dcl4* strain (Figure 1, Figure 4). The *pai1 dcl2 dcl3 dcl4* plants were larger and less fluorescent than *pai1* plants, reflecting the partial reduction of non-CG methylation levels on *PAI2* (Figure 4).

Examination of *pai1 dcl2 dcl3 dcl4* inbred populations revealed that blue fluorescence diagnostic of *PAI2* silencing was not stably maintained. In a population of 191 *pai1 dcl2 dcl3 dcl4* plants we found two non-fluorescent segregants (1.0%). In contrast, no non-fluorescent individuals were found in control populations of thousands of *pai1* plants, consistent with our previous results. Each of the non-fluorescent *pai1 dcl2 dcl3 dcl4* plants yielded approximately 75% non-fluorescent and 25% fluorescent second-generation progeny (54 non-fluorescent out of 70 total progeny plants [77%] for one line and 31 non-fluorescent out of 42 total progeny plants [74%] for another line). Approximately one third of the second-generation non-fluorescent plants lacked remaining *PAI2* 5meC in a *Hin*cII DNA gel blot assay, and these individuals yielded 100% non-fluorescent third-generation progeny, whereas the remaining second-generation non-fluorescent plants had partial levels of *PAI2* 5meC and yielded approximately 75% non-fluorescent third-generation progeny. For example, 12 out of 28 [43%] non-fluorescent second-generation progeny from one line had fully demethylated *PAI2* phenotypes in the *Hin*cII assay and each of these individuals yielded 100% non-fluorescent progeny; the *pai1 dcl* NF line shown in Figure 4 is derived from one of these individuals. The segregation patterns are consistent with reduced *PAI2* 5meC levels and increased expression occurring on just one of the two chromosomes in the parental non-fluorescent plant and being inherited in a Mendelian fashion.

DNA gel blot analysis of a non-fluorescent *pai1 dcl2 dcl3 dcl4* line indicated a nearly complete loss of CCG methylation monitored by *Msp*I cleavage and partially reduced CG methylation monitored by *Hpa*II cleavage at *PAI2* relative to the fluorescent parental line, consistent with the reversion of tryptophan deficiency phenotypes (Figure 4). However, *PAI1–PAI4* and *PAI3* maintained similar 5meC patterns to the fluorescent parental line. Therefore, in *pai1 dcl2 dcl3 dcl4* the loss of *PAI2* 5meC and silencing detected by the blue fluorescence screen is not coupled to destabilization of 5meC at the other *PAI* loci.

Both the initial loss of *PAI* non-CG methylation and the stochastic further loss of *PAI2* silencing and 5meC in *dcl2 dcl3 dcl4* are similar to patterns we previously observed in the *Δpai1–pai4* mutant [17]. This comparison indicates that regardless of whether *PAI* sRNAs are depleted by loss of DCL function or by loss of the source of *PAI* dsRNA substrates for DCL cleavage (Figure 3), *PAI2* silencing is similarly destabilized. The destabilization could be due to a combination of effects at *PAI2* including impairment of H3K9me2 maintenance, increased transcription, and impairment of the DRM2/DCL3 pathway in resetting 5meC imprints (Text S1, Figure S2).

### The *dcl2 dcl3 dcl4* mutant has reduced non-CG methylation levels at a subset of other SUVH/CMT3 target loci

**Figure 5:**
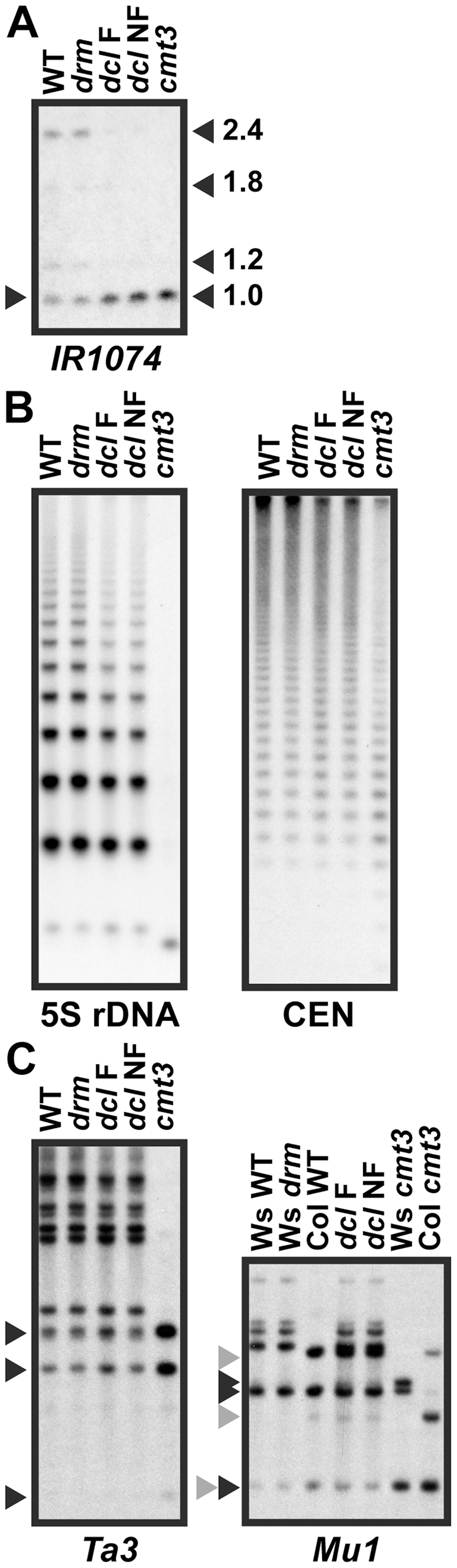
5meC levels are reduced at a subset of CMT3 target loci in *pai1 dcl2 dcl3 dcl4*. Genomic DNA from the indicated strains was cleaved with *Msp*I, or with *Msp*I plus *Hin*dIII for *Mu1*, and used in DNA gel blot analysis with the indicated probes. Arrowheads indicate positions of fully cleaved species. WT indicates wild type Ws. *drm* indicates the *drm1 drm2* double mutant. *dcl* F indicates a *pai1 dcl2 dcl3 dcl4* fluorescent strain and *dcl* NF indicates a *pai1 dcl2 dcl3 dcl4* non-fluorescent strain with *PAI2* demethylation, as shown in Figure 4. (A) *IR1074* is partially demethylated in *dcl* mutant strains (see Figure S1 for *Msp*I restriction map and probe information). (B) 5S rDNA and 180 bp centromere repeat sequences (CEN) are partially demethylated in *dcl* mutant strains. (C) *Ta3* and *Mu1* low-copy transposons are not demethylated in *dcl* mutant strains. For the *Mu1* blot, black and gray arrowheads indicate bands diagnostic of complete cleavage for the Ws or Col *Mu1* arrangements respectively.

To determine whether other SUVH/CMT3 target loci besides the *PAI* genes have reduced 5meC levels in the *dcl2 dcl3 dcl4* mutant, we used a survey approach with *Msp*I DNA gel blot assays (Figure 5). We monitored representative sequences of three types: a degenerate (86% identical) inverted repeat locus *IR1074* [29], highly repetitive 5S rDNA and 180 bp centromeric sequences (CEN), or low-copy transposons *Ta3* [7] and *Mu1* [30]. At all of these sequences, there was no difference in *Msp*I cleavage between wild type and the *drm1 drm2* mutant, but greatly increased cleavage in the *cmt3* mutant, indicating that CCG methylation at the monitored *Msp*I sites is dependent on CMT3 with a minimal contribution from the DRM MTases. For each sequence, we tested both the *pai1 dcl2 dcl3 dcl4* parental fluorescent strain and an isogenic non-fluorescent progeny line to determine whether these two strains had differences in 5meC patterns at loci other than *PAI2* (Figure 4).

The *IR1074*, 5S rDNA, and CEN sequences showed partially increased *Msp*I cleavage in both of the *dcl* mutant strains relative to wild type and *cmt3* (Figure 5). For the highly repetitive sequences the increased cleavage was evident as a slight shift downwards in the peak intensity of the ladder of cleaved bands (Figure 5B). Therefore, similarly to the *PAI* genes, these sequences require DCL function for maintenance of CMT3-dependent 5meC. The sequences had similar 5meC patterns in the fluorescent versus non-fluorescent *pai1 dcl2 dlc3 dcl4* lines, indicating that the loss of *PAI2* 5meC in the non-florescent line is not coupled to more general destabilization of 5meC.

In contrast, *Ta3* and *Mu1* showed no differences in *Msp*I cleavage between the *dcl* mutant lines and wild type controls (Figure 5). The *Mu1* transposon has a polymorphic arrangement between Ws and the Columbia (Col) strain in which the *dcl* alleles were originally isolated, and the *dcl2 dcl3 dcl4* strain carries both *Mu1* arrangements. Despite this complication, comparisons to wild type versus *cmt3* controls in each strain background showed no evidence of demethylation in the *dcl* mutant lines. Therefore, not all SUVH/CMT3 targets display DCL-dependent maintenance of 5meC. In light of our hypothesis that sRNAs prevent loss of H3K9me2 due to read-through transcription, the different effects of the *dcl* mutations at different SUVH/CMT3 target loci could reflect the extent to which read-through transcription occurs across the modified sequences. For example, the *dcl*-sensitive locus *IR1074* is likely to be transcribed across to make fold-back dsRNA because this locus produces sRNAs even in an RNA-dependent RNA polymerase *rdr2* mutant background [29].

**Figure 6:**
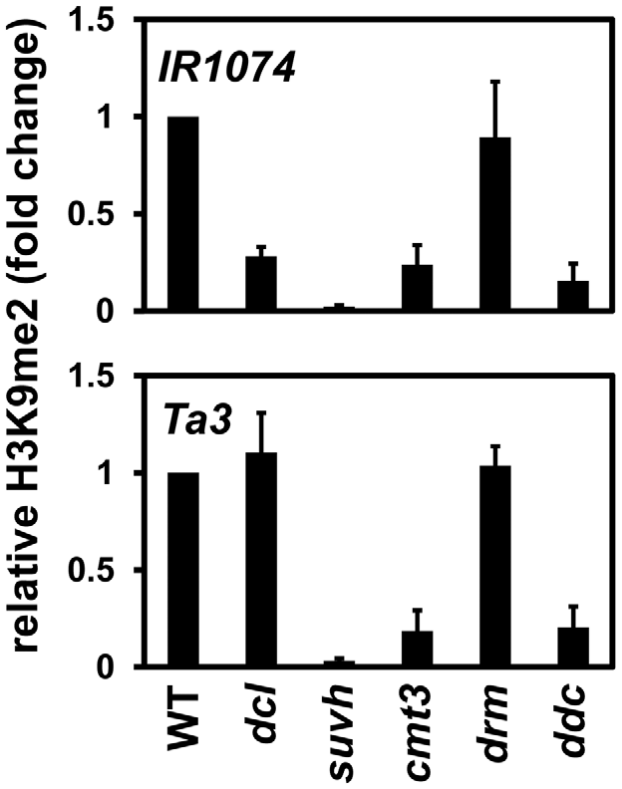
Maintenance of *IR1074* but not *Ta3* H3K9me2 patterns is impaired in *dcl2 dcl3 dcl4*. Primers specific for *IR1074* or *Ta3* were used for quantitative PCR amplification from total input chromatin or chromatin immunoprecipitated with antibodies specific for H3K9me2 from the indicated strains. WT indicates wild type Ws, *dcl* indicates the *dcl2 dcl3 dcl4* mutant, *suvh* indicates the *suvh4 suvh5 suvh6* mutant, *drm* indicates the *drm1 drm2* mutant, and *ddc* indicates the *drm1 drm2 cmt3* mutant. PCR results are displayed as fold change relative to WT. Results were reproduced in three biological replicates with error bars representing standard error among biological replicates.

We also monitored H3K9me2 levels at the single-locus targets *IR1074* and *Ta3* by ChIP in the *dcl2 dcl3 dcl4* mutant and the same control strains used for analysis of the *PAI* genes (Figure 6). At *IR1074* H3K9me2 levels were reduced in the *dcl2 dcl3 dcl4* mutant relative to wild type, although not as strongly as in the *suvh4 suvh5 suvh6* mutant. In contrast, at *Ta3* H3K9me2 was maintained at similar levels between the *dcl2 dcl3 dcl4* mutant and wild type. The H3K9me2 ChIP results agree with the 5meC results indicating that full modification of *IR1074* but not *Ta3* depends on DCL function (Figure 5A, Figure 5C).

The *cmt3* mutant had reduced levels of H3K9me2 at both *IR1074* and *Ta3*, presumably due to impaired SUVH localization and/or increased transcription caused by loss of non-CG methylation (Figure 5, Figure 6). However, at both loci the *drm1 drm2* mutant maintained H3K9me2 at similar levels to wild type, and the *drm1 drm2 cmt3* mutant maintained H3K9me2 at similar levels to *cmt3* (Figure 6). Therefore, CMT3 but not the DRM cytosine MTases contributes to maintenance of H3K9me2 patterns at *IR1074* and *Ta3*.

At *IR1074*, the *dcl2 dcl3 dcl4* mutant and the *cmt3* mutant displayed similar reductions in H3K9me2 levels relative to wild type (Figure 6). However, *dcl2 dcl3 dcl4* had a weaker reduction in *IR1074* non-CG methylation levels than *cmt3* (Figure 5). This relationship is similar to that observed for *PAI1–PAI4* and *PAI2* (Figure 1, Figure 2), and supports the view that loss of sRNAs in the *dcl2 dcl3 dcl4* mutant impairs maintenance of H3K9me2 patterns independently of CMT3 function at loci subject to read-through transcription.

## Discussion

In Arabidopsis, H3K9me2 maintained by the SUVH4, SUVH5, and SUVH6 histone MTases is used to guide 5meC in non-CG contexts maintained by the CMT3 cytosine MTase [1]–[4]. The SUVH MTases contain 5meC-binding domains, and CMT3 contains an H3K9me-binding domain, leading to the model that the SUVH/CMT3 pathway involves an amplification loop between 5meC and H3K9me2 [5], [6]. However, this amplification loop is not sufficient to maintain full levels of 5meC and H3K9me2 on the Ws *PAI* gene duplications, including a constitutively transcribed IR locus *PAI1–PAI4* and partially silenced singlet genes *PAI2* and *PAI3*. In previous work we showed that production of palindromic transcripts from the *PAI1–PAI4* IR is also required for maintenance of *PAI* non-CG methylation [13], [14], [17], [18]. For example, in a *Δpai1–pai4* mutant the *PAI2* and *PAI3* genes have reduced non-CG methylation, and the remaining 5meC on *PAI2* is destabilized upon inbreeding [17]. Here we use mutations in the DCL dicer ribonucleases to show that *PAI* sRNAs processed from *PAI* dsRNAs are the key species that reinforce the SUVH/CMT3 amplification loop between H3K9me2 and non-CG methylation.

Arabidopsis uses DCL-dependent sRNAs incorporated into argonaute (AGO) effector proteins as nucleic acid sequence-specificity guides in a variety of pathways including miRNA control of development, RNA interference, and guidance of 5meC mediated by the DRM2 cytosine MTase 19,23. The DRM2 pathway contributes together with the SUVH/CMT3 pathway to maintenance of non-CG methylation at many 5meC target loci [15], [31]. This overlap has obscured whether sRNAs have an independent role in the SUVH/CMT3 pathway. However, the Ws *PAI* genes maintain 5meC in non-CG contexts almost entirely through the SUVH/CMT3 pathway once initial 5meC is established (Figure 1, Text S1, Figure S2). The reduction in *PAI* non-CG methylation and H3K9me2 levels in *dcl* mutant backgrounds therefore indicates a direct connection between DCL-dependent sRNAs and the SUVH/CMT3 pathway (Figure 1, Figure 2). DCL2 and DCL3 are the key dicers required for maintaining H3K9me2 and 5meC patterns on the *PAI* genes, suggesting a preference for longer 22 and 24 nt sRNAs in this pathway.

ChIP analysis shows that the *dcl2 dcl3 dcl4* mutant has reduced H3K9me2 levels at *PAI* loci similar to or stronger than in the *cmt3* mutant (Figure 2). However, the *dcl2 dcl3 dcl4* mutant has weaker reductions in *PAI* 5meC levels than the *cmt3* mutant (Figure 1). Therefore reduced H3K9me2 levels in the *dcl* mutant cannot be accounted for as a secondary effect of impaired CMT3 function. Instead, the ChIP results support the view that the *dcl* mutations directly impair maintenance of H3K9me2 patterns. In this view, the partial loss of *PAI* H3K9me2 in *dcl2 dcl3 dcl4* reduces CMT3 localization, resulting in reduced *PAI* non-CG methylation as a secondary effect.

Similarly to the *PAI* genes, a subset of other SUVH/CMT3 target loci including highly repetitive 5S rDNA and CEN sequences have partially reduced non-CG methylation levels in the *dcl2 dcl3 dcl4* mutant (Figure 5). However, some loci such as the low copy transposons *Ta3* and *Mu1* can maintain full 5meC levels relative to wild type despite the loss of DCL function. This variation could reflect the degree to which different SUVH/CMT3 target loci are transcribed across. This variation could also reflect which RNA polymerases are most active at different loci. Arabidopsis encodes five RNA polymerases: the conserved eukaryotic RNA polymerases POLI, POLII, and POLIII, and plant-specific POLIV and POLV implicated in targeting DRM2-dependent 5meC [32]. In particular, POLV is proposed to transcribe across target loci to make “scaffold” transcripts that recruit sRNA/AGO complexes and components of the DRM2 pathway [33], [34]. Because of its specialized role in making silencing-associated transcripts, POLV might be less disruptive of H3K9me2 than other RNA polymerases designed to express host genes. In this case, protein-encoding loci like the *PAI* genes that are transcribed by RNA POLII, and 5meC targets that depend on RNA POLII for scaffold transcript synthesis [35], might have a stronger dependence on an sRNA-based mechanism to maintain H3K9me2 than POLV-transcribed regions of the genome.

Our previous studies with allelic variants of the *PAI1–PAI4* IR locus support the hypothesis that the level of transcription across the locus determines the extent to which sRNAs are needed for maintenance of *PAI* 5meC levels. In one study we used transgene-expressed sRNAs to direct 5meC and transcriptional silencing to the upstream promoter that drives transcription through *PAI1–PAI4*, thereby impairing production of *PAI* dsRNAs and sRNAs [14]. In this transgenic strain the *PAI1–PAI4* locus was able to maintain full 5meC levels, whereas the *PAI2* and *PAI3* singlet genes had partially reduced non-CG methylation levels. These patterns are consistent with a model where the decreased transcription of *PAI1–PAI4* specifically reduces its dependence on *PAI* sRNAs. In a second study we characterized a mutant derivative of Ws where a rearrangement in the center of the *PAI1–PAI4* IR introduces a new polyadenylation site and reduces the levels of transcripts that extend into palindromic *PAI4* sequences, without altering promoter sequences or the level of transcription across the locus [18]. In the rearrangement mutant the *PAI1–PAI4* IR locus as well as the *PAI2* and *PAI3* singlet genes had partially reduced non-CG methylation levels. These patterns are consistent with a model where read-through transcription at all three loci together with reduced *PAI* sRNAs results in loss of H3K9me2 and 5meC at all three loci, similarly to the situation in the *dcl2 dcl3 dcl4* mutant. Taken together, our results support a homeostatic mechanism where sRNAs produced from heterochromatic regions by read-through transcription feed back to counteract depletion of H3K9me2 and associated 5meC levels caused by read-through transcription.

The mechanistic relationship between sRNAs and maintenance of H3K9me2 patterns remains to be determined. In the fission yeast *Schizosaccharomyces pombe*, an sRNA-loaded AGO protein in the RITS effector complex interacts with nascent transcripts at centromeric repeats to recruit the Clr4 H3K9 MTase (reviewed in [36]). Plants could use an analogous effector complex interaction mechanism to target SUVH H3K9 MTases to specific regions of the genome. Consistent with this possibility, in a *suvh4 suvh5* mutant background, the remaining SUVH6 MTase maintains levels of H3K9me2 and associated 5meC similar to wild type at the *PAI1–PAI4* IR but not at the *PAI2* singlet gene [4]; this locus-specific activity could reflect preferential interactions between SUVH6 and effector complexes that assemble near a site of dsRNA synthesis. Alternatively, sRNA-AGO complexes could recruit intermediate factors that then promote SUVH activity at specific targets. A third possibility is that sRNAs could guide a pathway that protects heterochromatic sequences from H3K9 demethylation. For example, the IBM1 JumonjiC domain H3K9 demethylase acts to prevent H3K9me2 and non-CG methylation from accumulating in transcribed genes 11,37,38. IBM1 could be excluded from also acting at heterochromatic sequences through a mechanism that involves sRNA-AGO complexes. Furthermore, sRNA-dependent mechanisms that promote addition of H3K9me2 or prevent removal of H3K9me2 could operate in concert.

Pathways where sRNA-AGO complexes guide H3K9me to appropriate regions of the genome have been identified in organisms ranging from fission yeast to the protozoan *Tetrahymena thermophila* to the insect *Drosophila melanogaster*, even though these organisms lack 5meC [39]–[41]. Our discovery that Arabidopsis also uses sRNAs to maintain H3K9me could represent a plant-specific variation on this fundamentally conserved strategy. In this case, the sRNA/SUVH/CMT3 pathway and the sRNA/DRM2 pathway could have both evolved from a basal mechanism involving sRNA-AGO guidance of H3K9 MTases. Consistent with this possibility, SUVH variants that lack catalytic activity but maintain methyl-DNA binding are required for DRM2-dependent 5meC [42].

The plant sRNA/H3K9me maintenance mechanism is interwoven with the SUVH/CMT3 chromatin binding amplification loop and partially redundant functions of the MET1 and DRM pathways to create a reinforced silencing network. However, loss of the sRNA/H3K9me maintenance mechanism cannot be completely buffered by the other pathways, and results in both immediate reductions and longer-term destabilization of H3K9me2 and 5meC. The unique properties of the *PAI* genes make them ideal reporters to further understand how sRNAs are harnessed to control maintenance of H3K9me2 on appropriate target sequences in plant genomes.

## Materials and Methods

### Plant strains

T-DNA insertional *dcl* alleles were obtained from the Arabidopsis Biological Resource Center (ABRC) or from the laboratory of James Carrington at Oregon State University. The *dcl2-1*, *dcl3-1*, and *dcl4-2* mutations are likely null alleles originally isolated in the Col strain [21], [23]. The *dcl1-9* mutation is a partial function allele originally isolated in Ws, but then crossed five times to the Landsberg *erecta* (Ler) strain 24,25. Each *dcl* mutant was crossed to Ws. PCR-based genotype markers were used to identify *dcl* mutant progeny homozygous for the three *PAI* loci from Ws (Table S1). Each *dcl* allele was crossed a second time with Ws to increase the proportion of the genome contributed by the Ws parent. The resulting *dcl* single mutant strains were then crossed with each other to generate double, triple, and quadruple mutant combinations. The *dcl* mutations were also crossed into the Ws *pai1* reporter strain [28]. The Ws *drm1 drm2* double T-DNA insertional null strain was obtained from the laboratory of Steven Jacobsen at UCLA [43]. The Col *cmt3-11T* T-DNA insertional null strain was obtained from the ABRC [44]. The Ws *pai1*, Ws *Δpai1–pai4*, Ws *Δpai1–pai4*(*PAIIR*), Ws *cmt3i11a*, Ws x *met1-1*, and pai1 suvh4R302* suvh5-1 suvh6-1 strains were previously described [4], [14], [16], [17], [28]. Ws *cmt3illa* and Ws *drm1 drm2* mutants were crossed to make the Ws *drm1 drm2 cmt3* strain.

### Analysis of 5meC patterns

Plant genomic DNA preparation and DNA gel blot assays for 5meC were performed as previously described [12]. Bisulfite sequencing of the top strands of *PAI1* and *PAI2* proximal promoter regions was performed as previously described [14]. *PAI* bisulfite sequencing primers are listed in Table S1.

### sRNA analysis

Total RNA was extracted with TRIzol reagent (Invitrogen) using the manufacturer's protocol. Low molecular weight (LMW) RNA was enriched by precipitating high molecular weight RNA out of solution with 0.5 M NaCl, 10% polyethylene glycol (MW 8000). The remaining LMW RNA was precipitated with 100% ethanol and resuspended in water treated with diethyl pyrocarbonate. LMW RNA was fractionated on a 17% acrylamide 7 M urea gel and transferred to a Hybond-N membrane (GE Healthcare). sRNA 5′ ends were chemically crosslinked to the membrane as previously described [45]. Membranes were hybridized in OligoHyb buffer (Ambion) overnight at 42°C with ^32^P 5′ end-labeled oligonucleotide probes. Probes were either an LNA modified *PAI1* exon 5 sense 35-mer (Exiqon) or an *miR167* antisense 21-mer (Table S1). Probed membranes were washed three times with a 2× SSC, 0.1% SDS solution. sRNA sizes were estimated from an ethidium bromide-stained low molecular weight DNA ladder (USB), and by comparison to the *PAI* sRNA species observed in the *Δpai1–pai4*(*PAIIR*) control strain [14].

### ChIP analysis

Formaldehyde crosslinking and chromatin preparations were performed as previously described [46] starting with two grams of aerial tissue from three-week-old plants grown in soilless potting medium (Fafard mix 2) under continuous illumination. Chromatin was immunoprecipitated with anti-H3K9me2 monoclonal antibody [47] or carried through the protocol with no antibody added as a control (mock precipitation). Immunoprecipitations were performed as previously described [3]. Each ChIP assay was performed in at least three independent biological replicates. Quantitative PCR amplification of immunoprecipitated DNA was performed using the 7300 Real-Time PCR System (ABI), with three replicate reactions for each sample. ChIP primer sequences are listed in Table S1.

## Supporting Information

Figure S1 Restriction maps of loci analyzed by DNA gel blot. (A) *PAI* gene restriction maps. Arrows represent the regions of shared sequence identity among the duplicated *PAI* genes. Black arrows indicate duplications that share at least 98% identity and the gray arrow indicates that the *PAI3* duplication is more divergent, with only 90% identity to the other genes. The number of each *PAI* gene is listed on the arrowhead. The Ws *PAI* genes carry 5meC across the regions represented by the arrows. Restriction patterns unique to specific strains are indicated with Ws, Col, or Col/Ler. *Hin*cII sites are indicated by red vertical bars. *Msp*I/*Hpa*II sites are indicated by black vertical bars. Only the *Hin*cII and *Msp*I/*Hpa*II relevant to the 5meC DNA gel blot assays are shown. *PAI*-internal sites that are methylated in wild type Ws are indicated with asterisks. The regions covered by the probe used in *PAI* DNA gel blots are indicated by blue lines. The sizes of restriction products detected in DNA gel blot analysis are given in kb. The transcription start site (TSS) position for each *PAI* gene is indicated. (B) *IR1074* restriction map. Arrows represent the duplicated segments of the IR, with the degenerate nature of the duplication indicated with black versus gray arrows. *Msp*I sites are indicated by black vertical bars. Internal sites that are methylated in wild type Ws are indicated with asterisks. The region covered by the probe used in the *IR1074* DNA gel blot shown in Figure 5A is indicated by the blue line over the sequences flanking the right duplication. The sizes of restriction products detected in DNA gel blot analysis are given in kb.
(PDF)

Figure S2 The *dcl3* and *drm1 drm2* mutations impair acquisition of new *PAI2* 5meC. (A) Diagram of crossing scheme to test for initiation of *PAI2* 5meC and silencing. Col/Ler *PAI* genes are indicated by red arrows. Ws *PAI* genes are indicated by black or grey arrows, representing functional and non-functional genes respectively. 5meC is indicated by boxes around the affected genes. The question mark indicates that initiation of 5meC on *PAI2* depends on the genetic background. (B) DNA gel blot assay for *PAI* 5meC. Genomic DNA from the indicated strains in the indicated generations was cleaved with *Hin*cII and used in DNA gel blot analysis with a *PAI* cDNA probe. P1–P4 indicates *pai1–PAI4*, ColP1 indicates Col/Ler *PAI1*, and P3 indicates *PAI3*, with bands diagnostic of 5meC on *PAI*-internal sites marked with asterisks. P2 indicates unmethylated Ws, Col, or Ler *PAI2*, P2* indicates methylated Ws *PAI2*, and Col/LerP2* indicates methylated Col or Ler *PAI2*, which is a higher molecular weight species than in Ws due to a flanking *Hin*cII polymorphism (see Figure S1). Molecular weights of fragments are as shown in Figure 1. (C) Blue fluorescence diagnostic of *PAI2* silencing in the *pai1–PAI4* background. Representative 2.5-week-old plants of the indicated strains in the indicated generations are shown under visible (top) or UV (bottom) light. Ws *pai1* x Col/Ler indicates strains that are homozygous for Ws *pai1–PAI4* and homozygous for Col/Ler *PAI2*, as shown in (A), with the indicated *dcl* or *drm* mutations present in both parents. *drm* indicates the *drm1 drm2* mutant.
(PDF)

Table S1 Oligonucleotide sequences. Sequences for oligonucleotides used in this study are shown.
(PDF)

Text S1 The *dcl3* and *drm1 drm2* mutations impair acquisition of new *PAI2* 5meC. Results, Materials and Methods, and References supporting the experiments shown in Figure S2 are provided.
(PDF)
