# Supplementary material for: Small RNAs Prevent Transcription-Coupled Loss of Histone H3 Lysine 9 Methylation in Arabidopsis thaliana
Source: PLoS Genet. 2011 Oct 27;7(10):e1002350. doi: 10.1371/journal.pgen.1002350 (PMC3203196; doi:10.1371/journal.pgen.1002350)
Supplement: Figure S1 — Restriction maps of loci analyzed by DNA gel blot. (A) PAI gene restriction maps. Arrows represent the regions of shared sequence identity among the duplicated PAI genes. Black arrows indicate duplications that share at least 98% identity and the gray arrow indicates that the PAI3 duplication is more divergent, with only 90% identity to the other genes. The number of each PAI gene is listed on the arrowhead. The Ws PAI genes carry 5meC across the regions represented by the arrows. Restriction patterns unique to specific strains are indicated with Ws, Col, or Col/Ler. HincII sites are indicated by red vertical bars. MspI/HpaII sites are indicated by black vertical bars. Only the HincII and MspI/HpaII relevant to the 5meC DNA gel blot assays are shown. PAI-internal sites that are methylated in wild type Ws are indicated with asterisks. The regions covered by the probe used in PAI DNA gel blots are indicated by blue lines. The sizes of restriction products detected in DNA gel blot analysis are given in kb. The transcription start site (TSS) position for each PAI gene is indicated. (B) IR1074 restriction map. Arrows represent the duplicated segments of the IR, with the degenerate nature of the duplication indicated with black versus gray arrows. MspI sites are indicated by black vertical bars. Internal sites that are methylated in wild type Ws are indicated with asterisks. The region covered by the probe used in the IR1074 DNA gel blot shown in Figure 5A is indicated by the blue line over the sequences flanking the right duplication. The sizes of restriction products detected in DNA gel blot analysis are given in kb. (PDF) [file pgen.1002350.s001.pdf]

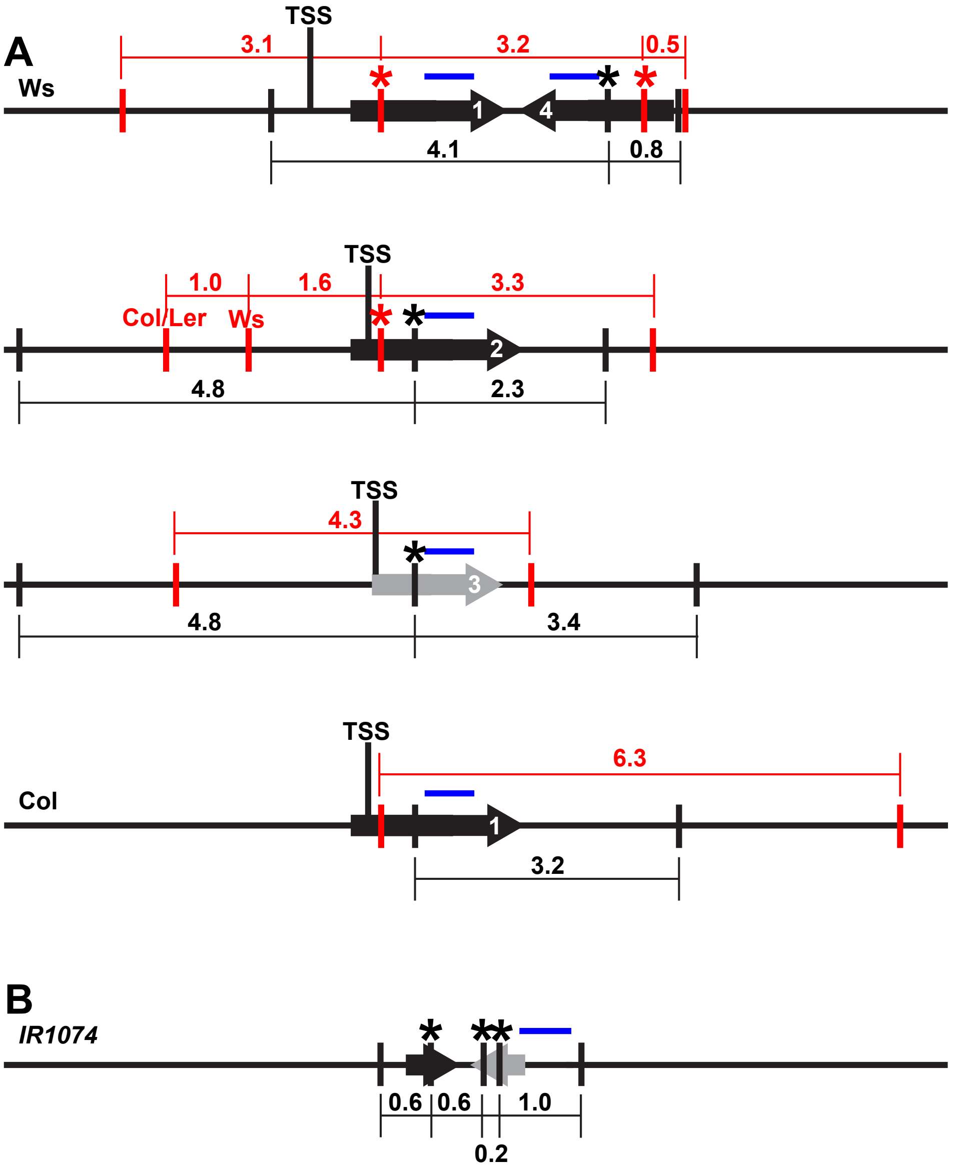

Figure S1. Restriction maps of loci analyzed by DNA gel blot.

(A) *PAI* gene restriction maps. Arrows represent the regions of shared sequence identity among the duplicated *PAI* genes. Black arrows indicate duplications that share at least 98% identity and the gray arrow indicates that the *PAI3* duplication is more divergent, with only 90% identity to the other genes. The number of each *PAI* gene is listed on the arrowhead. The Ws *PAI* genes carry 5meC across the regions represented by the arrows. Restriction patterns unique to specific strains are indicated with Ws, Col, or Col/Ler. *Hinc*II sites are indicated by red vertical bars. *Msp*I/*Hpa*II sites are indicated by black vertical bars. Only the *Hinc*II and *Msp*I/*Hpa*II relevant to the 5meC DNA gel blot assays are shown. *PAI*-internal sites that are methylated in wild type Ws are indicated with asterisks. The regions covered by the probe used in *PAI* DNA gel blots are indicated by blue lines. The sizes of restriction products detected in DNA gel blot analysis are given in kb. The transcription start site (TSS) position for each *PAI* gene is indicated. (B) *IR1074* restriction map. Arrows represent the duplicated segments of the IR, with the degenerate nature of the duplication indicated with black versus gray arrows. *Msp*I sites are indicated by black vertical bars. Internal sites that are methylated in wild type Ws are indicated with asterisks. The region covered by the probe used in the *IR1074* DNA gel blot shown in Figure 5A is indicated by the blue line over the sequences flanking the right duplication. The sizes of restriction products detected in DNA gel blot analysis are given in kb.
