# Supplementary material for: Small RNAs Prevent Transcription-Coupled Loss of Histone H3 Lysine 9 Methylation in Arabidopsis thaliana
Source: PLoS Genet. 2011 Oct 27;7(10):e1002350. doi: 10.1371/journal.pgen.1002350 (PMC3203196; doi:10.1371/journal.pgen.1002350)
Supplement: Figure S2 — The dcl3 and drm1 drm2 mutations impair acquisition of new PAI2 5meC. (A) Diagram of crossing scheme to test for initiation of PAI2 5meC and silencing. Col/Ler PAI genes are indicated by red arrows. Ws PAI genes are indicated by black or grey arrows, representing functional and non-functional genes respectively. 5meC is indicated by boxes around the affected genes. The question mark indicates that initiation of 5meC on PAI2 depends on the genetic background. (B) DNA gel blot assay for PAI 5meC. Genomic DNA from the indicated strains in the indicated generations was cleaved with HincII and used in DNA gel blot analysis with a PAI cDNA probe. P1–P4 indicates pai1–PAI4, ColP1 indicates Col/Ler PAI1, and P3 indicates PAI3, with bands diagnostic of 5meC on PAI-internal sites marked with asterisks. P2 indicates unmethylated Ws, Col, or Ler PAI2, P2* indicates methylated Ws PAI2, and Col/LerP2* indicates methylated Col or Ler PAI2, which is a higher molecular weight species than in Ws due to a flanking HincII polymorphism (see Figure S1). Molecular weights of fragments are as shown in Figure 1. (C) Blue fluorescence diagnostic of PAI2 silencing in the pai1–PAI4 background. Representative 2.5-week-old plants of the indicated strains in the indicated generations are shown under visible (top) or UV (bottom) light. Ws pai1 x Col/Ler indicates strains that are homozygous for Ws pai1–PAI4 and homozygous for Col/Ler PAI2, as shown in (A), with the indicated dcl or drm mutations present in both parents. drm indicates the drm1 drm2 mutant. (PDF) [file pgen.1002350.s002.pdf]

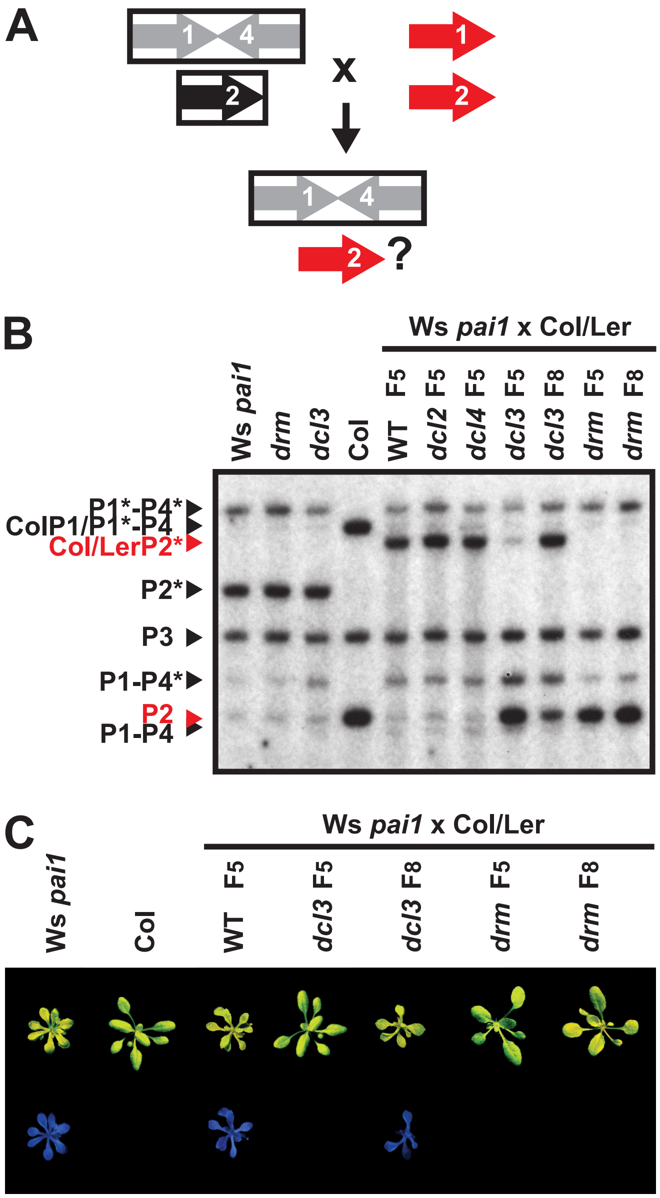

Figure S2. Initiation of *PAI2* 5meC and silencing in *dcl* and *drm* mutants.

(A) Diagram of crossing scheme to test for initiation of *PAI2* 5meC and silencing. Col/Ler *PAI* genes are indicated by red arrows. Ws *PAI* genes are indicated by black or grey arrows, representing functional and non-functional genes respectively. 5meC is indicated by boxes around the affected genes. The question mark indicates that initiation of 5meC on *PAI2* depends on the genetic background. (B) DNA gel blot assay for *PAI* 5meC. Genomic DNA from the indicated strains in the indicated generations was cleaved with *Hinc*II and used in DNA gel blot analysis with a *PAI* cDNA probe. P1-P4 indicates *pai1-PAI4*, ColP1 indicates Col/Ler *PAI1*, and P3 indicates *PAI3*, with bands diagnostic of 5meC on *PAI*-internal sites marked with asterisks. P2 indicates unmethylated Ws, Col, or Ler *PAI2*, P2* indicates methylated Ws *PAI2*, and Col/LerP2* indicates methylated Col or Ler *PAI2*, which is a higher molecular weight species than in Ws due to a flanking *Hinc*II polymorphism (see Figure S1). Molecular weights of fragments are as shown in Figure 1. (C) Blue fluorescence diagnostic of *PAI2* silencing in the *pai1-PAI4* background. Representative 2.5-week-old plants of the indicated strains in the indicated generations are shown under visible (top) or UV (bottom) light. Ws *pai1* x Col/Ler indicates strains that are homozygous for Ws *pai1-PAI4* and homozygous for Col/Ler *PAI2*, as shown in (A), with the indicated *dcl* or *drm* mutations present in both parents. *drm* indicates the *drm1 drm2* mutant.
