# Supplementary material for: Small RNAs Prevent Transcription-Coupled Loss of Histone H3 Lysine 9 Methylation in Arabidopsis thaliana
Source: PLoS Genet. 2011 Oct 27;7(10):e1002350. doi: 10.1371/journal.pgen.1002350 (PMC3203196; doi:10.1371/journal.pgen.1002350)
Supplement: Table S1 — Oligonucleotide sequences. Sequences for oligonucleotides used in this study are shown. (PDF) [file pgen.1002350.s003.pdf]

Table S1. Oligonucleotide sequences.

locus	sequence (5'-3')	probe/product size
dcl1-9 T-DNA	KFLB : GATGCACTCGAAATCAGCCAATTTTAGAC<br>DCL1F2: CTGTGGAAGTTTTCATCGACGGTG	~450 bp
DCL1 intact gene	DCL1F2<br>DCL1R2: CCAATTAAACTCGAATAACAACAGATC	637 bp
dcl2-1 T-DNA	SALKLB : GCGTGGACCGCTTGCTGCAACT<br>DCL2F2: GGCTGAGATACCTCAAGGTGGTTT	~600 bp
DCL2 intact gene	DCL2F2<br>DCL2R3: CCTCTCCGGAAGTCTTCCACAATT	576 bp
dcl3-1 T-DNA	SALKLB<br>DCL3F1: CCTGAAGAGCGTGAAGGAGTGG	~700 bp
DCL3 intact gene	DCL3F1<br>DCL3R2: CCTTGCACACCATTGAGCATTC	584 bp
dcl4-2 T-DNA	GABILB : ATATTGACCATCATACTCATTGC<br>DCL4-2F: GGCTGCACAGCTGATGATTACAA	~600 bp
DCL4 intact gene	DCL4-2F<br>DCL4-2R: GCCGCTCGAGATCATCAGCAAAGGAAT	908 bp
drm1-1 T-DNA	JL202: CATTTTATAATAACGCTGCGGACATCTAC<br>DRM1TR: CTTCCATACTTGCATCTTCTC	~400 bp

DRM1 intact gene	DRM1TF: CGAATGCACTTCTAACTTATGCG<br>DRM1TR	604 bp
drm2-1 T-DNA	JL202<br>DRM2TR: CTCTCCTTTTGACTCAAACCGCC	~350 bp
DRM2 intact gene	DRM2TF: CTTTAGCTGTCGAGAGATGTG<br>DRM2TR	785 bp
PAI1 Col/Ler vs. Ws genotype	PAI1AF: GGTACAATTGATCTTCACTATAG<br>PALPR: CCTCTACTAAACAAGTCAAAC	Col/Ler: 395 bp<br>Ws: 425 +410 bp
PAI2 Col vs. Ws/Ler genotype	CER456052F: TGGCGTAATGTTCGTGAA<br>CER456052R: CAAAATGAGTATCCTTAAC	Col: 200 bp<br>Ws/Ler: 165 bp
PAI2 Col/Ws vs. Ler genotype	CER454289F: CACACCTGTGGAGCCCAAG<br>CER454289R: CACGCGCCAAAAGTGCGTG	Col/Ws: 210 bp<br>Ler: 250 bp
PAI3 Col vs. Ws/Ler genotype	PAI3F: CTTATTATGACATTCGTTCTGCTCG<br>PAI3R: CTCTTTGATCTCGCCATGAGCACC	after *Mnl*I digest:<br>Col/Ler: 300 bp<br>Ws: 170 + 130 bp
PAI1 proximal promoter bisulfite sequencing	P1TF: GCTCTAGATGYAGAATTYTGTGYATTTG<br>P1TR: CGGGATCCTRTTRACATCTTAATTTCAC	500 bp
PAI2 proximal promoter bisulfite sequencing	P2TF: GCTCTAGATTAATGTTTYGAAGATGATAAG<br>P1TR	486 bp
PAI1 ChIP	PAI1CHIPEF: TAGAGGATTGAGCTTAAGGCAAGAT<br>PAI1CHIPGR: TTCAAGTCCCCGATGTTCTTTA	125 bp
PAI2 ChIP	P2I1BF: TCAGTTAATGAAACAAGCTTTG	75 bp

	P2E2DR: TTCAGAGCTCCAAAATGGACG	
IR-1074 ChIP	1074CHIPF: TCCAGTTGGCTTAGCTTCACCAGAACC<br>1074CHIPR: CTCGCTCTCTTTTCCTCTCAGAGAACCT	115 bp
Ta3 ChIP	TA3F: AGACAGCTCTGCGTGGAAGTC<br>TA3R: TTATCAGTCTCAGCATTACACAGTT	90 bp
miR167 antisense probe	TAGATCATGCTGGCAGCTTCA	21 nt
PAI1 exon 5 LNA sense probe	GgGGgAATcAATcCAAcAAATgTTTcAGAAg CTcT (lower case= LNA residues)	35 nt
IR1074 probe	IR1074F1: GGTTCTCTGAGAGGAAAAGAGAGC<br>IR1074R1: GCCCAATATCGCTCTTAATTGGGTTC	780 bp
