# Supplementary material for: Small RNAs Prevent Transcription-Coupled Loss of Histone H3 Lysine 9 Methylation in Arabidopsis thaliana
Source: PLoS Genet. 2011 Oct 27;7(10):e1002350. doi: 10.1371/journal.pgen.1002350 (PMC3203196; doi:10.1371/journal.pgen.1002350)
Supplement: Text S1 — The dcl3 and drm1 drm2 mutations impair acquisition of new PAI2 5meC. Results, Materials and Methods, and References supporting the experiments shown in Figure S2 are provided. (PDF) [file pgen.1002350.s004.pdf]

Supporting Information

The *dcl3* and *drm1 drm2* mutations impair acquisition of new *PAI2* 5meC

To test the effects of *dcl* mutations in establishing new *PAI* 5meC imprints triggered by the *PAI1-PAI4* transcribed IR we used a previously developed assay (Figure S2). In this assay, a *pai1-PAI4* missense mutant variant of the 5meC trigger locus is combined by crossing with a naïve unmethylated target *PAI2* gene from a strain that lacks a *PAI1-PAI4* IR such as Columbia (Col) or Landsberg *erecta* (Ler) [1,2]. Because the *PAI3* and *PAI4* genes contain polymorphisms that inactivate enzyme function [3], in the resulting hybrid strain the target *PAI2* gene is the only source of fully functional PAI enzyme. Therefore *PAI2* 5meC-mediated transcriptional silencing can be detected by acquisition of a PAI-deficient blue fluorescent phenotype caused by accumulation of the tryptophan precursor anthranilate. In a wild type background, the *pai1-PAI4* transcribed IR triggers accumulation of 5meC and reduced expression of the target *PAI2* gene sufficient for blue fluorescence by the F_2 or F_3 (S_1) generation. The 5meC density on *PAI2* triggered by the *PAI* IR locus increases progressively over subsequent generations of inbreeding by self-pollination [4].

We performed a similar assay combining *pai1-PAI4* and unmethylated *PAI2* in hybrids where both parents carried single *dcl2*, *dcl3*, or *dcl4* mutations. Independent hybrids generated in the *dcl2* or *dcl4* mutant backgrounds (two lines for each genotype) acquired blue fluorescence by the F_2 generation, similarly to hybrids generated in a wild type background. The blue fluorescence in the *dcl2* and *dcl4* hybrids corresponded to acquisition of 5meC on the target *PAI2* gene, as assessed by *Hinc*II DNA gel blot assays on DNA from F_5 (S_3) generation plants (Figure S2). In contrast, none of six independent hybrid lines generated in the *dcl3* mutant background displayed blue fluorescence in the F_2 generation, and none of these lines segregated blue

fluorescent progeny until the F_6 (S_4) generation. *Hinc*II DNA gel blot analysis of *dcl3* hybrids indicated that non-fluorescent F_5 (S_3) plants carried a low level of *PAI2* non-CG methylation whereas fluorescent F_8 (S_6) plants carried *PAI2* non-CG methylation at a similar density to fluorescent wild type, *dcl2*, or *dcl4* hybrids. These results indicate that DCL3 is the major dicer involved in initiation of *PAI* 5meC, but that other dicers can contribute at a less efficient level in the absence of DCL3.

We also used the hybrid plant assay to test the effects of the *drm1 drm2* mutations on initiation of *PAI2* 5meC imprints. None of six independent *drm1 drm2* hybrid lines segregated blue fluorescent individuals or displayed detectable *PAI2* non-CG methylation as assessed by *Hinc*II DNA gel blot even by the F_8 (S_6) generation (Figure S2). These results indicate that DRM1/DRM2 are required for initiation of *PAI2* 5meC, even though they are dispensable for maintaining existing *PAI* 5meC (Figure 1). In a previous study we found that a *cmt3* mutation also prevents initiation of *PAI2* 5meC in the hybrid plant assay [1]. Taken together, our results support the view that DRM1/DRM2 initiate a low level of *PAI* 5meC, which is then amplified and maintained through the SUVH/CMT3 and MET1 pathways.

Materials and Methods for Supporting Information

Assay for initiation of *PAI2* 5meC

To analyze the effects of *dcl* mutations on initiation of *PAI2* 5meC imprints, the Col allele of *dcl2-1*, *dcl3-1*, or *dcl4-2* was crossed with the same allele in the Ws *pai1* background. F_2 progeny were scored with PCR-based genotype markers to find individuals homozygous for the Ws *pai1-PAI4* locus and homozygous for the Col *PAI2* locus (Table S1). For *dcl2* and *dcl4* two independent lines with the desired genotype were analyzed for each cross. For *dcl3* and *drm1 drm2*, six independent lines with the desired genotype were analyzed for each cross. In each subsequent generation of each inbred F_2 line, progeny from two independent plants were assessed for blue fluorescence under UV light. Because the *drm1 drm2* alleles were originally isolated in Ws, we performed a series of crosses to move these alleles into a background where the *PAI2* gene had not been even transiently exposed to Ws *PAI1-PAI4*: 1) Ws *drm1 drm2* was crossed with Col, and F_2 plants were scored with PCR-based genotype markers to find *drm1 drm2* individuals homozygous for Col *PAI1* and Col *PAI2*, 2) three F_2 individuals with the desired genotype from the first-round cross were each crossed with Ler, and F_2 plants were scored with PCR-based genotype markers to find *drm1 drm2* individuals homozygous for Ler *PAI2*, 3) one F_2 individual with the desired genotype from each second-round cross was crossed with a Ws *pai1 drm1 drm2* strain, and F_2 plants were scored with PCR-based genotype markers to find individuals homozygous for the Ws *pai1-PAI4* locus and homozygous for the Ler *PAI2* locus, and 4) two individuals with the desired genotype from each of the three third-round crosses were inbred by self-pollination for a total of six independent lines.

References for Supporting Information

1. Malagnac F, Bartee L, Bender J (2002) An *Arabidopsis* SET domain protein required for maintenance but not establishment of DNA methylation. EMBO J 21: 6842-6852.

2. Melquist S, Bender J (2004) An internal rearrangement in an Arabidopsis inverted repeat locus impairs DNA methylation triggered by the locus. Genetics 166: 437-448.

3. Melquist S, Luff B, Bender J (1999) Arabidopsis *PAI* gene arrangements, cytosine methylation and expression. Genetics 153: 401-413.

4. Luff B, Pawlowski L, Bender J (1999) An inverted repeat triggers cytosine methylation of identical sequences in *Arabidopsis*. Mol Cell 3: 505-511.

5. Bender J, Fink GR (1995) Epigenetic control of an endogenous gene family is revealed by a novel blue fluorescent mutant of *Arabidopsis*. Cell 83: 725-734.
